# New Mexico’s Capacity for Increasing the Prevalence of Colorectal Cancer Screening With Screening Colonoscopies

**Published:** 2004-12-15

**Authors:** Richard M Hoffman, S. Noell Stone, Carla Herman, Ann Moore Jung, Jane Cotner, David Espey, Richard Kozoll, Michael W Gavin

**Affiliations:** New Mexico VA Health Care System; University of New Mexico Cancer Center, Albuquerque, NM; University of New Mexico Cancer Center, Albuquerque, NM; New Mexico Medical Society, Albuquerque, NM; University of New Mexico Cancer Center, Albuquerque, NM; Indian Health Services, Centers for Disease Control and Prevention, Albuquerque, NM; New Mexico Medical Society, Cuba, NM; Lovelace Sandia Health System, Albuquerque, NM

## Abstract

**Introduction:**

Colorectal cancer screening rates are low throughout the United States. Colonoscopy has been recommended as a cost-effective strategy for colorectal cancer screening and prevention. We evaluated New Mexico's capacity to increase the prevalence of colorectal cancer screening using colonoscopy.

**Methods:**

We identified New Mexican gastroenterologists from state licensing data and from endoscopic manufacturers. We surveyed gastroenterologists on their weekly number of colonoscopies, capacity for additional screening colonoscopies, and barriers to increasing capacity. We used census data, published data on the yield of screening colonoscopy, and professional society guidelines for cancer/polyp surveillance to estimate the additional colonoscopies required to increase the state's prevalence of endoscopic screening.

**Results:**

Forty gastroenterologists, representing all 11 group practices in the state, and nine of 12 solo practitioners responded. They estimated that their weekly procedure capacity could be increased by 41%, from 832 to 1174 colonoscopies. We estimated an annual capacity increase of 14,880 procedures, which could increase the prevalence of endoscopic colorectal cancer screening from the current 35% to about 50% over five years. Lack of support staff, space, and physicians were barriers to increasing screening.

**Conclusion:**

Implementing a screening colonoscopy strategy could achieve the goal of a higher level of colorectal screening. However, achieving more universal screening would require additional testing modalities.

## Introduction

Colorectal cancer is the third most frequently diagnosed cancer in New Mexico and the second leading cause of cancer death (1). Randomized controlled trials of fecal occult blood testing (FOBT) have shown that screening reduces the incidence and mortality of colorectal cancer (2-4). Flexible sigmoidoscopy has also been shown to reduce colorectal cancer mortality in well-designed case-control studies (5,6). Professional organizations have identified colorectal cancer screening as an effective, high-priority intervention (7-10). Acceptable modalities include FOBT, flexible sigmoidoscopy, colonoscopy, and double-contrast barium enema.

Despite those supportive practice guidelines, colorectal cancer screening rates remain low. National data show that just over 50% of adults aged 50 years and older are considered to be appropriately screened for colorectal cancer with either a FOBT within one year or an endoscopic procedure within 10 years (11,12). In New Mexico, 2001 survey data from the Centers for Disease Control and Prevention's (CDC's) Behavioral Risk Factor Surveillance System (BRFSS) reported that only 23.2% of adults aged 50 years and older had undergone FOBT testing in the previous two years and that 34.5% had undergone a flexible sigmoidoscopy or colonoscopy in the previous five years (13). The BRFSS survey did not obtain information about radiographic screening tests. Overall, only 48% of the adult population was considered currently screened (analysis by the New Mexico BRFSS Unit, July 2003); this likely is an overestimate given the limited concordance of the BRFSS colorectal cancer screening questions with medical records (14) and the potential selection bias introduced by the telephone survey design.

Efforts to improve screening rates have included celebrity endorsements by Katie Couric (15), the CDC's Screen for Life campaign (16), the American Cancer Society's "Polyp Man" public service announcements (17), and President Clinton's 2000 declaration that March would henceforth be National Colorectal Cancer Awareness Month (18). Medicare began reimbursing for colorectal cancer screening with FOBT and flexible sigmoidoscopy in 1998 and has reimbursed screening colonoscopy at 10-year intervals for average-risk adults since July 2001 (19). The National Center for Quality Assurance has established a new Health Plan Employer Data and Information Set measure of colorectal cancer screening performance standards for health care plans beginning in 2004 (20,21).

Although there is no direct evidence for its screening efficacy, colonoscopy is the most accurate diagnostic test and offers the potential to remove premalignant growths. Winawer and colleagues estimated that colonoscopy could reduce the incidence of colorectal cancer by a range of 76% to 90% (22). Economic analyses have also found that colonoscopy is a cost-effective screening strategy for colorectal cancer (23-25). The American College of Gastroenterology practice guidelines recommend colonoscopy to be the first screening option (26). However, experts have questioned the feasibility of increasing screening through colonoscopy because the number of colonoscopists and infrastructure needed to screen the population may be inadequate (9). We conducted a survey of New Mexican gastroenterologists to determine the feasibility of implementing a colonoscopic screening strategy to improve statewide screening rates.

## Methods

The Colorectal Cancer Screening Working Group of the Clinical Prevention Initiative (CPI) evaluated screening capacity by conducting a mailed survey of endoscopists in New Mexico. The CPI membership, composed of public health and health care professionals, is supported by the New Mexico Department of Health and the New Mexico Medical Society to promote more effective delivery of practice-based preventive services throughout the state of New Mexico.

### Subjects

We identified endoscopists in New Mexico by using data from the Board of Medical Examiners, contacting manufacturers of endoscopic equipment, and obtaining the membership lists of a statewide gastroenterology journal club, the New Mexico Medical Society, and the American Medical Association. Eligible subjects for this analysis were gastroenterologists actively practicing in New Mexico, which included 40 gastroenterologists practicing in one of the 11 group practices and 12 solo practitioners.

### Survey

The CPI colorectal cancer group developed a brief survey to obtain information about endoscopic capacity, including colonoscopies and flexible sigmoidoscopies (Table 1). Questions were based on literature review, the BRFSS, and the clinical experience of the CPI colorectal cancer group, which included two gastroenterologists and two internists who performed sigmoidoscopy. Revisions were based on pilot testing the survey with clinical colleagues and other members of the CPI. The survey was conducted between October and December 2001. Subjects were mailed a letter introducing the survey and asking for their participation. The survey was printed on a postcard with a return address and postage. For nonrespondents, we followed up with telephone calls and repeat mailings two weeks after the initial contact.

### Statistical analysis

We used simple, descriptive nonparametric statistics to estimate the weekly median number of procedures performed by endoscopists in group practice and solo practice and the estimated weekly potential increase in capacity.

**Endoscopic capacity.** We determined the number of additional screening colonoscopies that could be performed using survey responses. We averaged responses when multiple members of a group practice completed the survey and provided different estimates for the weekly number of baseline and additional procedures performed by the practice. We imputed the weekly number of baseline and additional colonoscopies for the solo-practitioner nonrespondents using data from the responding solo practitioners. For the annual number of colonoscopies, we assumed that endoscopists performed procedures for 40 weeks. We performed similar estimates for the number of flexible sigmoidoscopies.

**Volume of colonoscopies.** We modeled the number of procedures required for a statewide screening colonoscopy strategy. To identify the number of subjects potentially eligible for colonoscopic screening, we used data from the 2000 United States Census for New Mexico that reported 468,000 resident adults aged 50 to 85 (27). Based on the census data, we evaluated the additional number of screening colonoscopies required to increase the prevalence of current screening by 5% (23,400 additional people being screened), 10% (46,800), 15% (70,200), 20% (93,600), and 25% (117,000) during a five-year period. We assumed that the additional screening procedures would be performed in equal numbers during the five-year period. We then modeled the number of surveillance procedures that would be required following the initial screening colonoscopy. We used clinical data on the yield of colorectal cancers and adenomatous polyps from a recent large Department of Veterans Affairs (VA) colonoscopic screening trial (28) and consensus guidelines for the timing of surveillance procedures (10).

- Colorectal cancer detection level: 1%
- Adenomatous polyp detection level: 37%
- Advanced (villous, dysplastic, >1 cm, >2 polyps) polyp level: 15%
- Surveillance following colorectal cancer detection: 6 months and 3 years
- Surveillance following 1–2 adenomatous polyps <10 mm: 5 years
- Surveillance following advanced polyp: 3 years

We assumed that half of the cancers diagnosed in the fifth year would have a six-month surveillance colonoscopy that same year. The colonoscopic screening trial had a higher proportion of subjects with positive family history of colorectal cancer than the general population and may have overestimated the yield of screening. Results from an employee-health colonoscopic screening program did show a lower yield than the VA study (29). Therefore, we performed a sensitivity analysis by reducing the expected rates of detected colorectal cancers and adenomatous polyps by approximately 50%.

We entered survey data into a Microsoft Access (Microsoft Corporation, Seattle, Wash) database. We performed statistical analyses with SAS (SAS Institute, Inc, Cary, NC) (30).

## Results

We received procedure information from nine of 12 solo practitioners and all 11 group practices, representing 40 endoscopists (two to eight practitioners per group). Physicians and practices were based in 12 different counties. Ten of 11 group practices and six of 12 solo practitioners were located in urban areas, defined by the Census Bureau as having population densities >1000 per square mile (31). Table 2 shows the numbers of procedures currently being performed weekly and the weekly capacity for additional procedures, which were stratified by type of practice. Overall, gastroenterologists reported performing 832 colonoscopies a week; they estimated being able to increase their capacity by an additional 342 (41%) procedures each week.

Assuming a 40-week work year, each endoscopist in group practice could perform an estimated 252 additional colonoscopies every year and solo practitioners could perform an estimated 400 additional colonoscopies. Statewide, endoscopists could perform an estimated 13,680 additional colonoscopic procedures each year. If the nonresponding solo practitioners performed similarly to those completing the survey, the estimated annual additional capacity for colonoscopy would be 14,880 procedures.

We show the estimated number of additional colonoscopies required to increase screening prevalence by 5%, 10%, 15%, 20%, and 25% during a five-year period in Table 3. The total number of procedures includes screening procedures based on the 2000 New Mexico census and surveillance procedures based on the yield of cancer and adenomatous polyps detected with screening. The second column of numbers reflects the yield of advanced neoplasia based on the VA study data from Lieberman and colleagues. The third column is a sensitivity analysis showing the estimated number of colonoscopies if the cancer yield was 0.5% and the overall yield of adenomatous polyps was 20%. If all patients with adenomatous polyps underwent colonoscopic surveillance at three years (rather than just patients with advanced neoplasia), the annual number of procedures would be increased by about 5%. Overall, a screening colonoscopy strategy could increase the prevalence of current colorectal cancer screening by about 15%.

Although our analyses focused on colonoscopies, we also obtained information on flexible sigmoidoscopy. All but one of the group practices performed flexible sigmoidoscopies, but only five of the solo practitioners performed them. Overall, however, only 165 procedures were performed weekly; respondents estimated that they could perform an additional 188 procedures.

The barriers to performing additional endoscopic tests are shown in Table 4. Only one group practice reported no barriers to performing additional procedures, and four solo practitioners reported no barriers. Lack of support staff, space (for procedures and/or recovery room), and physicians were the most frequently cited problems for the group and solo practices.

## Discussion

New Mexico gastroenterologists responding to our survey estimated having the capacity to increase their weekly number of colonoscopies by about 41%, from 832 to 1174. This substantial increase could raise the prevalence of current endoscopic screening by approximately 15% within five years. The most recent BRFSS data report that 35% of New Mexican adults are currently screened by endoscopy; thus, the increased endoscopic capacity would be just sufficient to achieve 50% colorectal cancer screening. However, this level of screening would still be far short of the 70% to 90% screening reported for mammography, Papanicolaou (Pap) smears, and prostate-specific antigen (PSA) tests (32,33). Additional recommended screening modalities, including FOBT, flexible sigmoidoscopy, and radiological studies would be needed to achieve a higher level of screening (7,8).

Rex and Lieberman modeled a strategy for implementing colonoscopy as the preferred screening procedure in the United States (9). Based on a 10-year screening interval and assuming that 10% of the adult population aged 50 to 70 would be screened every year, they estimated an annual need for 7.7 million colonoscopies. After reducing this number for patients with significant comorbidities, noncompliance, and current screening, they estimated that approximately 2.56 million additional colonoscopies would need to be performed. Based on a government report that 4.4 million colonoscopies were performed in 1999, Rex and Lieberman concluded that implementing screening colonoscopy would require a 58% increase in capacity. This figure may be an underestimation because they modeled screening only until age 70. Given that the incidence of colorectal cancer increases steadily with age (34) and that screening could appropriately be offered until age 80 (35), the actual number of additional colonoscopies could be quite higher.

Even if Rex and Lieberman correctly estimated the number of additional procedures to fully implement screening colonoscopy, the demand in New Mexico would likely exceed the capacity of the state's endoscopists — despite their already high level of productivity. Endoscopists in New Mexico reported performing about 16 to 20 colonoscopies weekly, which compares quite favorably with data obtained from the National Cancer Institute's (NCI's) nationwide Survey of Colorectal Cancer Screening Practices. The 346 gastroenterologists responding to the survey, conducted between November 1999 and April 2000, performed an average of only 31.7 colonoscopies monthly, including 12.4 for screening (36).

Rex and Lieberman acknowledged that increasing the level of colonoscopies would be challenging (9). One of their solutions was for gastroenterologists to perform 50% fewer flexible sigmoidoscopies to make time to perform colonoscopies (9). They cited Medicare data showing that 543,502 flexible sigmoidoscopies were performed in 2000. The nationwide NCI survey estimated that gastroenterologists performed 25% of sigmoidoscopies, which suggests that nearly 70,000 fewer procedures could be performed in just the Medicare population alone (36). However, this survey indicated that sigmoidoscopies comprised about 30% of the colorectal endoscopic procedures performed by gastroenterologists. Our data showed that sigmoidoscopy comprised only 16% of the lower endoscopic procedures performed by gastroenterologists in New Mexico, implying practice patterns had already changed substantially. Further reductions in performing sigmoidoscopy may not be feasible, especially because many of the sigmoidoscopic procedures are diagnostic.

Another strategy for implementing screening colonoscopy would be to increase the number of procedures performed by other medical providers. The NCI survey reported that general surgeons performed 30% of colonoscopies (36). However, on average, the 251 general surgeons performed fewer than eight colonoscopies monthly, including about three screening colonoscopies. While nearly half of the gastroenterologists performed at least 10 screening colonoscopies monthly, only 6% of general surgeons reached this level. The NCI survey also obtained data on colorectal cancer screening practices by primary care physicians (37). Among the 1235 respondents, fewer than 5% of primary care providers reported performing colonoscopy, and most of them performed fewer than five procedures monthly. Although 29% of primary care respondents performed sigmoidoscopy, fewer than 20% performed more than 10 procedures monthly.

Increasing screening colonoscopy by having general surgeons and primary care physicians perform these procedures does not seem to be a feasible strategy for New Mexico. When we conducted our survey, endoscopic equipment manufacturers provided us information on all practices that had purchased equipment for performing colorectal procedures. In addition to gastroenterologists, we also identified surgeons and primary care physicians as owners of endoscopic equipment. Three of the eight colorectal cancer surgeons in the state identified as performing colonoscopies responded to the survey; they were performing 18 colonoscopies weekly and estimated that they could increase their capacity by 12 weekly. None of the 28 primary care providers who performed endoscopy reported performing colonoscopy. Only six primary care endoscopists reported performing five or more (maximum eight) flexible sigmoidoscopies weekly; the majority performed less than two. Another problem with relying on nongastroenterologists to perform endoscopy is that their low procedure volume may be associated with diminished proficiency (38).

Rex and Lieberman further noted that increasing capacity for screening colonoscopy would require more efficiency in endoscopy suites (9). Our respondents consistently reported that limited space and support staff were barriers to performing more procedures. Our respondents also reported that having more physicians would help improve capacity. Strategies to increase the number of gastroenterologists would likely target a training program; New Mexico does have a university gastroenterology fellowship program. However, Rex and Lieberman questioned the wisdom of training more endoscopists because accurate, cost-effective, noninvasive tests — such as virtual colonoscopy or fecal DNA assays — are likely to be used increasingly, thus reducing the need for screening colonoscopies (9). Another barrier facing New Mexico is a relatively impoverished population with a high proportion of uninsured adults (39); financial incentives may also be necessary to attract and retain specialists. Although there is little data supporting the practice, nonphysicians could also be trained to perform colonoscopy (40).

Our study had some important limitations. We were generally unable to validate the self-reported weekly number of procedures performed by each practice or solo practitioner. However, one group of three gastroenterologists, who estimated that they annually performed 3000 procedures, also reviewed their billing records for the previous year. These data showed that they had overestimated their current capacity by 10% — they actually performed only 2760 procedures. The estimated increased capacity also depends upon the respondents being able to accurately assess the practice's ability to perform additional tests, which could not be validated. However, if other practices similarly overestimated their current capacity, then the estimates for the absolute number of additional procedures could also be inflated.

Another potential limitation was that we used a simplified model. On the demand side, we assumed that patients would be compliant with surveillance-testing recommendations and that the population would be stable. On the supply side, we assumed that the number of gastroenterologists in the state would be stable. We also assumed that the supply of endoscopists would be matched with patients needing procedures. However, New Mexico has problems retaining specialists (41), and even having a sufficient number of gastroenterologists may not ensure comprehensive screening coverage. In New Mexico, access to care may be severely limited by geographic distance. New Mexico is a large, mostly rural state; almost all of the gastroenterologists practice in urban areas. Nonetheless, our intention was not to precisely estimate screening capacity but rather to provide a general assessment of the feasibility of implementing screening colonoscopy, including identifying provider barriers.

We conclude that New Mexico has the colonoscopic capacity to substantially increase the prevalence of adults with current colorectal cancer screening. The state could probably achieve a level of 50% current endoscopic screening by colonoscopy alone. However, New Mexico lacks the capacity to implement a fully comprehensive screening colonoscopy strategy. Efforts to achieve more universal screening would also require additional modalities such as FOBT, flexible sigmoidoscopy, and barium enema in addition to health care policies requiring screening coverage. More efficient use of colonoscopy would also be necessary, including withholding colonoscopic screening from patients with limited life expectancy (7), performing surveillance colonoscopy at appropriate intervals (42), and considering a single lifetime-screening colonoscopy strategy (43).

## Figures and Tables

**Table 1:** Questions for Survey of Gastroenterologists on Colonoscopy Screening Capacity, New Mexico, 2001

1. How many endoscopists work in your practice?					
2. How many perform colonoscopy?					
3. How many perform sigmoidoscopy?					
4. During an average week, how many colonoscopies do you perform?					
5. During an average week, how many colonoscopies are performed in your practice?					
6. During an average week, how many sigmoidoscopies do you perform?					
7. During an average week, how many sigmoidoscopies are performed in your practice?					
8. How many additional screening colonoscopies could your practice perform in a week?					
9. How many additional screening sigmoidoscopies could your practice perform in a week?					
10. What resources would be required to perform additional endoscopic procedures?					
None	More equipment	More space	More support staff	More physicians	Other

**Table 2:** Current Volume of Colonoscopies Performed Weekly and Weekly Capacity for Additional Colonoscopies, New Mexico, 2001

**Practice Type**	**Total number of endoscopists**	**Current colonoscopies per endoscopist^a^**	**Total current colonoscopies**	**Additional colonoscopies per endoscopist^a^**	**Total additional colonoscopies**
Group	40	16.3 (12.9, 26.5)	652	6.3 (1.8, 10)	252
Solo	9	20 (15, 21)	180	10 (5, 15)	90
Combined	49	NA^b^	832	NA	342

a Values are median (interquartile range).

b NA = not applicable.

**Table 3:** Number of Colonoscopies Required to Increase the Prevalence of Current Screening During a Five-year Period for New Mexico Adults Aged 50 to 85 Years

**Screening increase over five years (%)**	**Annual number of colonoscopies (based on detection rates from VA study)^a^**	**Annual number of colonoscopies (based on detection rates from sensitivity analysis)^b^**
5	5568 (5983)^c^	5137 (5360)
10	11,136 (11,966)	10,274 (10,721)
15	16,704 (17,949)	15,411 (16,082)
20	22,272 (23,932)	20,548 (21,442)
25	27,840 (29,915)	25,568 (26,800)

a Includes numbers of screening tests based on 2000 New Mexico census data and numbers of surveillance tests based on applying cancer (1.0%) and adenomatous polyp (37%) detection rates from a Department of Veterans Affairs (VA) study (28).

b Includes numbers of screening tests based on 2000 New Mexico census data and numbers of surveillance tests based on applying cancer (0.5%) and adenomatous polyp (20%) detection rates from sensitivity analysis.

c Numbers in parentheses reflect the strategy of performing a three-year surveillance colonoscopy on all patients with adenomatous polyps compared to five-year surveillance interval.

**Table 4:** Barriers to Performing Additional Endoscopic Tests, Results of a Survey of Gastroenterologists, New Mexico, 2001^a^

**Barrier**	**Group practices** **N = 11**	**Solo practitioners** **N = 9**
**None**	1	4
**More equipment**	4	2
**More space**	8	1
**More support staff**	7	3
**More physicians**	8	4
**Other (lack of referrals)**	1	0

a More than one barrier could be reported.
