# Erratum, Vol. 2: No. 1

**Published:** 2005-09-15

In the PDF version of the article “New Mexico’s Capacity for Increasing the Prevalence of Colorectal Cancer Screening With Screening Colonoscopies,” Table 4 (of four tables) was omitted. The table, “Barriers to Performing Additional Endoscopic Tests, Results of a Survey of Gastroenterologists, New Mexico, 2001,” did, however, appear in the HTML version. The PDF version was corrected on our Web site on August 12, 2005. It appears online at http://www.cdc.gov/pcd/issues/2005/jan/04_0073.htm. We regret any confusion this error may have caused.

